# Developing a Model for Pharmaceutical Palliative Care in Rural Areas—Experience from Scotland

**DOI:** 10.3390/pharmacy5010006

**Published:** 2017-02-16

**Authors:** Gazala Akram, Emma Dunlop Corcoran, Alison MacRobbie, Gill Harrington, Marion Bennie

**Affiliations:** 1 Strathclyde Institute of Pharmacy and Biomedical Sciences, University of Strathclyde, Glasgow G4 0RE, UK; emma.d.corcoran@strath.ac.uk (E.D.C.); Marion.Bennie@strath.ac.uk (M.B.); 2 NHS Highland, Inverness IV2 3BW, UK; alison.macrobbie@nhs.net; 3 The Boots Company PLC, 5 Wentworth St, Portree IV51 9EJ, UK; gillian.harrington1@nhs.net

**Keywords:** pharmaceutical palliative care, community pharmacy, medication used in palliative care, rural, service evaluation, support staff, education and training

## Abstract

Palliative care is increasingly delivered in the community but access to medicines, particularly ‘out of hours’ remains problematic. This paper describes the experience of developing a model to deliver pharmaceutical palliative care in rural Scotland via the MacMillan Rural Palliative Care Pharmacist Practitioner (MRPP) project. The focus of the service was better integration of the MRPP into different care settings and professional teams, and to develop educational resources for the wider MDT including Care Home and Social Care staff on medicine related issues in palliative care. A variety of integration activities are reported in the paper with advice on how to achieve this. Similarly, many resources were developed, including bespoke training on pharmaceutical matters for Care Home staff. The experience allowed for a three step service and sustainability model for community pharmacy palliative care services to be developed. Moving through the steps, the key roles and responsibilities of the MRPP gradually shift towards the local Community Pharmacist(s), with the MRPP starting from a locality-based hands-on role to a wider supportive facilitating role for local champions. It is acknowledged that successful delivery of the model is dependent on alignment of resources, infrastructure and local community support.

## 1. Introduction

Palliative care is increasingly delivered in the community setting [1,2,3]. However a lack of forward or anticipatory care planning often means that unnecessary problems such as ‘problematic and unsatisfactory’ access to medicines, especially to unfamiliar, unlicensed or Controlled Drugs can become an issue [4,5]. Better communication between care settings or services, and inclusion of the pharmacist (specialist or community) in medicine related decisions can potentially avoid some of these problems [6,7]. In 2009, NHS Greater Glasgow and Clyde Health Board (NHS GG and C) in partnership with Macmillan Cancer Support, appointed four part-time Macmillan Pharmacist Facilitators (NHS GG and C is one of the largest health boards in Scotland covering a population of approximately 1.2 million and containing 112 community pharmacies). The facilitator posts were designed ‘to improve the provision of pharmaceutical palliative care services through better engagement with local community pharmacies’. The success of the Glasgow facilitator service model [5,8] highlighted the potential of pharmacy in improving palliative care service delivery. With this in mind, a collaboration between NHS Highland Health Board, Macmillan Cancer Support and The Boots Company PLC set out to develop community pharmacy capacity for providing pharmaceutical palliative care to a rural population in the Scottish Highlands through the funding of 1 (full time) Palliative Care Pharmacist Practitioner. This paper describes the experiences of developing and delivering such a service in a remote and rural part of the north west of Scotland. This area (known as the Isle of Skye and the Kyle of Lochalsh) was chosen because it is a clearly defined area and district within the Highland council area with recognised boundaries reflecting a rural community with geographic challenges. The area included dispensing GPs, community pharmacies, district nursing services, Macmillan nursing services, community hospitals, care homes and care at home services. There are also smaller island populations nearby which require access to the Island of Skye for health services within the area.

The MacMillan Rural Palliative Care Pharmacist Practitioner (MRPP) project was delivered in two phases from 2013 to 2015. Phase 1 was concerned with characterising the current provision of community pharmacy palliative care services. Phase 1 utilised a mixed case study approach comprising of questionnaires, interviews, audits and documentary data gathered through engagement with GPs, patients, carers, Key Service Personnel or Leads (KSLs), Care Home staff/management, to identify gaps in current service provision that could potentially be met by the MRPP [9]. The overarching theme to emerge from this Phase was that local health professionals had a poor understanding of the wider (clinical) role of community pharmacists (i.e., beyond a medicines supply function) and correspondingly limited expectations. This influenced the decision that Phase 2 should concentrate on raising the profile of ‘pharmacy’ in the delivery of pharmaceutical palliative care amongst local patients and the wider multidisciplinary team (MDT). The focus would be on achieving integration of the MRPP into different care settings/teams and on developing opportunities for education/training about medicines used in palliative care. In doing so, a variety of quality improvement and engagement activities were applied across the project locale.

## 2. Materials and Methods 

### 2.1. Study Population and Setting 

The project covered a land mass area approx 2700 km^2^ with a population of 13,238. The Isle of Skye covers 1.6 km^2^ with estimated population density of 6 people per km^2^. Approximately 35% of the population are aged between 45–64 years with 20% over 65 years. Patients in this highly rural area can receive medication from one of three community pharmacies or from one of the four GP dispensing practices. The largest District General Hospital is approximately 2 h drive from the most eastern location and 2.5 h drive from the main town at the outer western limits. The distance between the furthest two villages in the project area takes approximately 2 h in the car and covers almost 84 miles. The populations of the towns and villages in the project area are diverse. The most densely populated area has approx. 1900 patients registered with one GP practice, compared with the least dense area, which has a registered patient population of 280. Further details are provided in Table 1.

### 2.2. Programme Evaluation Approach 

The MRPP undertook a variety of activities over the project time period (January 2014 to December 2015). These included: attendance and contribution at MDT meetings in various settings; development of clinics within the community pharmacy setting; design and dissemination of pharmaceutical palliative care educational resources and training sessions.

A qualitative approach was employed to capture service user’s experiences of the MRPP role, assess its impact and highlight areas for further development. Eight of the 12 interview participants from Phase 1 (KSLs drawn from a number of areas including Nursing, General Practice, Social Work, Scottish Ambulance Service and NHS Management) agreed to a follow-up telephone interview 12 months from project commencement. In addition, one patient and carer also shared their experiences of the MRPP. Furthermore, the activities of the MRPP were captured and documented through an oral history approach whereby the researcher called the MRPP on a regular basis over a 21 week period during 2014 to generate a chronological timeline for the MRPP role (11 occasions in total). The MRPP would detail their recent activities and an unstructured conversation would ensue.

All stakeholder interviews were between 10–40 min long, digitally recorded with hand written notes also taken by the researcher. The interviews were transcribed using an intelligent verbatim approach and a thematic analysis undertaken [11]. One researcher read all of the transcripts (with one transcript each being read by two other researchers to ensure validation). A list of themes was compiled and further refined/validated through peer consensus on a sample of the interviews. This revised framework was applied to the remainder of the transcripts and refined as appropriate. Transcription was done using Microsoft Word with analysis performed by NiVivo 9 (QSR International, Melbourne, Australia) a qualitative data analysis software.

The oral histories were audio recorded and typed in note form to enable documentation of the MCPP activities.

NHS ethical approval was sought for the project but as it was a Service Evaluation, a full ethics application was deemed unnecessary. However, all aspects of the process were adhered to including maintaining patient/client anonymity and consent for participation.

## 3. Results (Project Phase 2)

### 3.1. Implementation of MRPP Role 

The stakeholder analysis identified several areas detailing the MRPPs engagement with the wider MDT and patients. One of the KSLs identified that initially engagement was challenging, as existing teams for a variety of reasons were perhaps not open to expanding their established networks. Furthermore, it seemed that challenging the pre-existing and traditional view of the pharmacist was difficult but paramount to the successful integration of the role into the wider palliative care team. Most participants reported that the MRPP’s knowledge of palliative care medicines was the most helpful aspect for them. Some healthcare professionals saw the MRPP as a source of information, or at the very least, a signpost to information that they required. Many of the participants stated that the MRPP had been particularly helpful in situations where sourcing a medicine had been problematic:

“ [This patient’s] medicine was trying to keep symptoms under control … [the MRPP] was able to [phone] the company that was producing it as well as talk to other pharmacists for advice for what other things we could try for him.”

*(KSL)*

The participants also noted that prior to the MRPP role, local GPs or the Highland Hospice were often their first port of call when seeking information about (palliative) medicines but not anymore.

“it has been helpful having her there, because I think we turn to her more because before … [we] used to ring the hospice. And I know the one lady that we used to get in touch with there, they made her redundant. Her post was taken away*. So [the MRPP’s] filled a gap for us” (KSL) *

*(postholder retired-post not filled)*

“If you asked each of us GPs I think we would say ... collectively … there’s been positive merits from all of my colleagues about her involvement”

*(GP)*

The MRPP was also active in the local Community Hospitals, auditing and monitoring medicines use that further raised their profile:

“In the hospital pharmacy there are certain drugs that we have to have available 24/7 and there are other medicines that we use as standard medications … [the MRPP] assisted the nurse in organising that…She also helped by scrutinising prescriptions within the hospital and that’s a role a pharmacist in a hospital would normally take … to go through the prescriptions and see what the doctors are ordering and point out you know discrepancies or errors or suggestions given her particular knowledge on medications.”

*(KSL)*

Most participants commented on the MRPP’s involvement with patients and her role in terms of guiding and supporting palliative patients and their medicines. One participant reported that as the MRPP role was community based, it meant that care was more accessible:

“[The MRPP] talks about more than just medicines … I think it is something that's missing [elsewhere] to be honest with you, I believe it's that personal care, where a healthcare professional can go into a patient's home and really look after them in a rounded way, but have the expertise of the medicines that will help them through their condition and help them understand how to take them.”

*(KSL)*

Some participants gave specific examples of where the MRPP had directly helped a particular patient with their medicines, both in terms of improving access and how to take them. One participant, a family carer, detailed how the MRPP helped her while caring for her husband.

“[The MRPP] phoned up and made an appointment … she was quite helpful in looking carefully at the medication [my husband] was on and making suggestions … that was helpful because when you're a carer on your own, you can't start mucking about with medication … [the MRPP’s] input was good in that it gave me the confidence that I was doing the right thing and that I could also contact her if I was anxious about anything.”

*(Carer)*

Participants also referred to an array of care settings in which the MRPP had interacted with patients, including patient’s own homes, local Care Homes, hospitals and even in the street.

“She’s going round all the [care] homes doing a lot of education which for me is hugely beneficial … we can prevent a lot of symptom management problems later on and also get [the Care Homes] to contact us earlier when there is a problem”

*(Nurse)*

Some commented that the MRPP was increasingly becoming known in the community and had forged relationships with healthcare staff and had become personally and professionally known to many patients and family carers.

**Figure 1:**
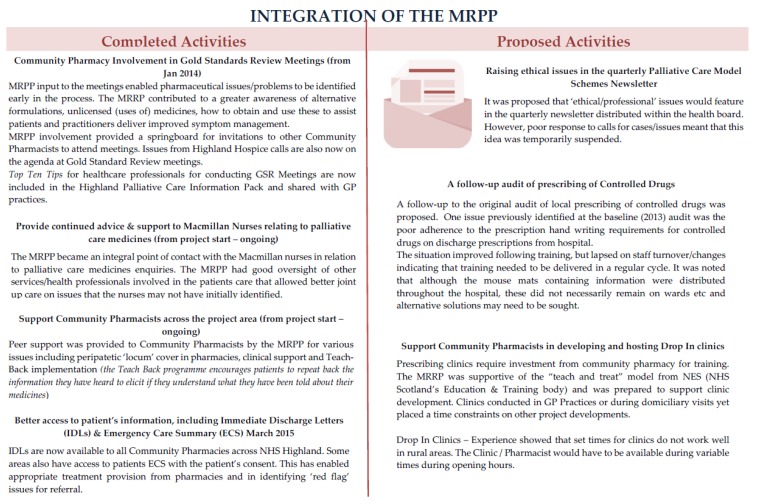
Integration of MacMillan Rural Palliative Care Pharmacist Practitioner (MRPP) Work Areas.

The oral histories largely described the MRPP involvement with the Gold Standards Review (GSR) meetings and other clinical duties. The GSRs are attended by a variety of healthcare and social care professionals who make decisions about a patient’s care in the various settings. The MRPP was often one of the few healthcare professionals who could “follow” these patients as they moved from location to location, and could therefore promote a more joined-up service. GP Dispensing Practices did not have GSR meetings prior to the introduction of the MRPP role but over time, the range of healthcare professionals attending the GSR meetings increased, possibly buoyed by a better awareness of the role of pharmacy and how this could impact on better care of the patient. The MRPP however reported that attending GSRs could be challenging due to staffing issues (at their community pharmacy base which made it difficult to leave the premises), timing of meetings (which were often at lunchtime) and travel time (up to 30–45 min). Other clinical services the MRPP had been instrumental in delivering included a ward based pharmacy service in the largest of the two Community Hospitals and a new medication ordering scheme in the other. The MRPP commented that over time, she was more welcome and accepted as part of the hospital team and that staff expected to see her there on a regular basis. The MRPP had also began informally training nursing staff in medicines ordering/management protocols and on a more specialist level, offered informal training specifically on palliative care medicines. See Figure 1 for a detailed explanation of activities.

### 3.2. Education and Training

**Figure 2:**
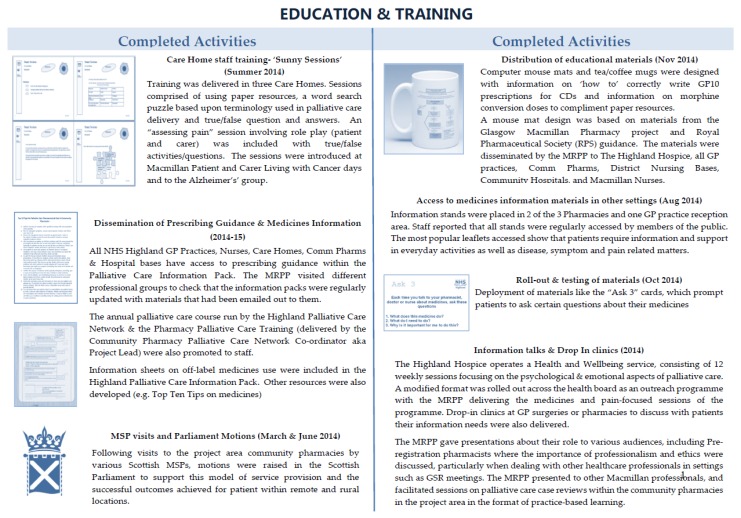
Education and Training Work Areas.

The MRPP was involved in the design and development of a number of educational materials as illustrated in Figure 2.

For example, an earlier audit of prescriptions for controlled drugs (CDs) presented for dispensing (on GP10 prescription forms) at the three community pharmacies showed errors were common in the writing of CD prescriptions. Good practice education materials to resolve issues with the incorrect writing of CD prescriptions were subsequently developed and distributed. Other materials focused on supporting patients and their carers on how to use their medicines more effectively, e.g., the Ask 3 cards and by the delivery of face to face training, called “Sunny Sessions” for healthcare staff working in Care Homes.

Of 16 Care Home staff who had undertaken the face to face training and completed an evaluation questionnaire, the majority (*n* = 14, 88%) were Care Assistants or Social Care workers with most (*n* = 12) working full-time. They were asked to rate their satisfaction of the training using a 5 point Likert scale (“Strongly Disagree” to “Strongly Agree”) to attitudinal statements. The majority responded that the face-to-face training approach, delivered during work hours in the work environment was the preferred method of delivery. All participants agreed that the training was useful, informative, well delivered and appropriate for untrained care home staff to increase their awareness of palliative care and what it entails. One participant identified that prior to the training, they weren’t aware of the full extent of planning that went into preparing for a patient’s end of life and ensuring the patient and their family’s wishes were met. Another participant said the training had increased her awareness of proper mouth care for palliative patients who may be experiencing issues associated with certain medications.

## 4. Discussion

The MRPP programme has succeeded in raising the profile of the pharmacy profession amongst not only the palliative care workforce but also amongst local patients and other health professionals in this rural community. Once the concept of the new service was understood, implementation was easier, and it became a feasible option for supporting enhanced utilisation of existing services and development of new ones. Evidence to support the claim that the MRPP role has had positive impacts on service efficiency, safety, effectiveness and was patient-centred and implemented in a timely manner can be measured by some of the resulting observed changes to local practice. By example, Community Pharmacy access to Immediate Discharge Letters (IDL) across NHS Highland is a real achievement given the positive impact this can have on patient care. Community Pharmacists have previously failed to receive timely information when a patient has been discharged from hospital and of any subsequent changes to the medication [12,13]. A hard copy of the discharge prescription, is usually the only means of communication between the hospital and the GP practice. In Scotland, a central online secure repository now exists (Scottish Care Information (SCI) store [14]) which allows relevant third parties access to ‘point of care’ patient information e.g., patient demographics, lab reports, some clinical information and details of hospital admissions/transfers and discharge. Access to the SCI store now allows community pharmacists to provide more effective pharmaceutical (palliative) care. An outcome of the MRPP service is that Caldicott Guardian approval for community pharmacy access to IDLs has been granted. Defining the key data areas and other governance issues (i.e., access for locums) required careful negotiation but proved worthwhile.

A second key impact of the MRPP role has been the adaptation of the ‘Sunny Session’ training to help inform a national education and training resource. During the lifetime of the MRPP project, a variety of government actions plans called on better delivery of palliative care and identified education and training of health and social care staff as priority areas [15,16]. The potential of the Sunny Sessions for wider education/training purposes including all health and social care staff was realised by the programme team. Led by NHS NES (NHS Scotland’s Education and Training body) a national online Palliative Care training series for health and social care support staff has since been funded in collaboration with NHS GG and C, Macmillan Cancer Support and the University of Strathclyde. The online training was developed and piloted amongst Care Home staff, GP receptionists and community pharmacy support staff and was launched nationwide in November 2016 via the NES online portal.

**Figure 3:**
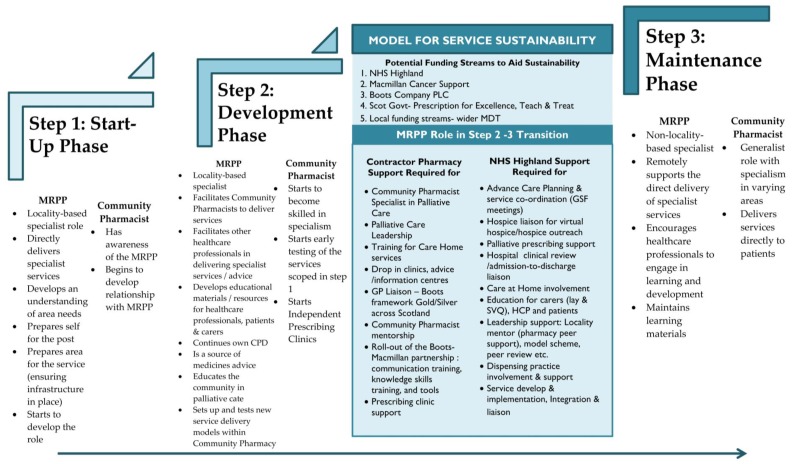
Step Diagram of Project Phases for Sustainability of Service.

To support evolving rural services, Figure 3 details a potential model for delivery of pharmaceutical palliative care services that is composed of three essential steps: Start-Up, Development and Maintenance. Moving through the steps the key roles and responsibilities of the MRPP gradually shift towards the local Community Pharmacist(s), with the MRPP graduating from locality-based hands on role to a more regional-based supporting and facilitating role for local champions. It was acknowledged that successful delivery of the model is dependent on alignment of resources, infrastructure and local community support. Sustainability of the service is perhaps the most important aspect. The addition of this domain puts into context current policy/funding drivers and key factors (in this case for NHS Scotland) necessary for delivery of pharmaceutical palliative care services. The three elements of this sustainability domain are:

- Policy/Funding—a range of potential funding streams/bodies that could be approached to financially aid the transition between Step 2 and Step 3.
- Community pharmacy—the support required to facilitate transfer of responsibilities from the MRPP to other pharmacy services within the community.
- Health Board—the support required to facilitate transfer of responsibilities from the MRPP to other professionals/services within the wider community.

Moving forward, the overarching aims of the MRPP project seem fully aligned with the current direction for Palliative and Urgent Care in Scotland. Progress is being made; transition of some services using the locally negotiated model for pharmaceutical palliative care service specification has been implemented. For example, Community pharmacists whose patients are to be discussed at Gold Standard Framework meetings are now routinely invited as part of the multidisciplinary team, which is a lasting legacy. The future ambition is for a 2 day-per-week co-ordinator role for an agreed geography to maintain and evolve service continuity including extension of education and training programmes and improved integration across health and social care, not currently included within the community pharmacy contract. Continual development is restricted without funding given the difficulties faced by community pharmacists to leave the premises to deliver additional services which although may bring value for patients and their families, e.g., visiting and supporting patients and family carers in their own homes, are likely to have an impact on their business. Opportunistic access to the MRPP role is limited at this time; this informal access to the post holder played a more critical role for patient and carer access than initially realised or intended.

## 5. Conclusions 

In conclusion, the impact of the service is still being felt in the community, partly due to the presence and availability of the training tools and resources developed which have been widely distributed. Furthermore, the expected versus preferred and actual route of access to the MRPP and the services they facilitated provided insight into the needs of the community, i.e., a more hands-on, person-focused and convenience-based service seems most suitable for this remote community.

## Figures and Tables

**Table 1:** Heath service provision in project area [10].

	Health Board Area NHS Highland (*n*)	Project Area of Skye, Kyle and Lochalsh (*n*)
Population	320,000	13,238
Land mass	32,500 km^2^	2700 km^2^
GP practices	392	26 (includes 4 dispensing practices)
Community Pharmacies	78	3 (5 pharmacists, excluding the MRPP)
District Nursing teams	24	2
Community Hospitals	10	2
Care Homes	17	6 (reduced to 4 in 1st year)
